# Long-term survival after self-expanding metallic stent or stoma decompression as bridge to surgery in acute malignant large bowel obstruction

**DOI:** 10.1093/bjsopen/zrab018

**Published:** 2021-04-21

**Authors:** T Axmarker, M Leffler, M Lepsenyi, H Thorlacius, I Syk

**Affiliations:** Department of Surgery, Skåne University Hospital, Malmö, Sweden; Department of Clinical Sciences, Malmö, Lund University, Malmö, Sweden; Department of Surgery, Skåne University Hospital, Malmö, Sweden; Department of Clinical Sciences, Malmö, Lund University, Malmö, Sweden; Department of Surgery, Skåne University Hospital, Malmö, Sweden; Department of Clinical Sciences, Malmö, Lund University, Malmö, Sweden; Department of Surgery, Skåne University Hospital, Malmö, Sweden; Department of Clinical Sciences, Malmö, Lund University, Malmö, Sweden; Department of Surgery, Skåne University Hospital, Malmö, Sweden; Department of Clinical Sciences, Malmö, Lund University, Malmö, Sweden

## Abstract

**Aim:**

Self-expanding metallic stents (SEMS) as bridge to surgery have been questioned due to the fear of perforation and tumour spread. This study aimed to compare SEMS and stoma as bridge to surgery in acute malignant large bowel obstruction in the Swedish population.

**Method:**

Medical records of patients identified via the Swedish Colorectal Cancer Register 2007–2009 were collected and scrutinized. The inclusion criterion was decompression intended as bridge to surgery due to acute malignant large bowel obstruction. Patients who underwent decompression for other causes or had bowel perforation were excluded. Primary endpoints were 5-year overall survival and 3-year disease-free survival. Secondary endpoints were 30-day morbidity and mortality rates.

**Results:**

A total of 196 patients fulfilled the inclusion criterion (SEMS, 71, and stoma, 125 patients). There was no significant difference in sex, age, ASA score, TNM stage and adjuvant chemotherapy between the SEMS and stoma groups. No patient was treated with biological agents. Five-year overall survival was comparable in SEMS, 56 per cent (40 patients), and stoma groups, 48 per cent (60 patients), *P* = 0.260. Likewise, 3-year disease-free survival did not differ statistically significant, SEMS 73 per cent (43 of 59 patients), stoma 65 per cent (62 of 95 patients), *P* = 0.32. In the SEMS group, 1.4 per cent (one patient) did not fulfil resection surgery compared to 8.8 per cent (11 patients) in the stoma group (*P* = 0.040). Postoperative complication and 30-day postoperative mortality rates did not differ, whereas the duration of hospital stay and proportion of permanent stoma were lower in the SEMS group.

**Conclusion:**

This nationwide registry-based study showed that long-term survival in patients with either SEMS or stoma as bridge to surgery in acute malignant large bowel obstruction were comparable. SEMS were associated with a lower rate of permanent stoma, higher rate of resection surgery and shorter duration of hospital stay.

## Teaser

This is a nationwide registry-based study on acute malignant large bowel obstruction comprising 196 cases in which all medical files were scrutinized. The results show that SEMS as bridge to surgery were associated with comparable long-term survival and recurrence rate compared to decompression by stoma, higher rate of fulfilled resection surgery and lower permanent stoma rate.

## Introduction

Colorectal cancer is the third most common malignancy globally, with more than 1.8 million new cases reported in 2018^1^. Approximately 15 per cent of large bowel cancers present as emergencies due to large bowel obstruction^2^^,^^3^. Emergency resection for malignant large bowel obstruction is associated with high perioperative morbidity and mortality rates^4–8^, with a reported postoperative morbidity rate of up to 51 per cent^4^^,^^9^ and 30-day mortality varying between 8 per cent and 13 per cent^2^^,^^9^^,^^10^. Hence, a two-step procedure has been advocated as an alternative to emergency resection in an attempt to decrease the morbidity rate. In this setting, placement of self-expanding metallic stents (SEMS) has been introduced, serving as bridge to surgery to overcome the high morbidity associated with emergency resection and decrease the need for stomas. However, the initial enthusiasm was attenuated after early reports of a high risk of perforation^11^^,^^12^ carrying a subsequent increased risk of recurrence^13^^,^^14^. Moreover, concerns have been raised regarding the increased risk of haematogenous spread due to trauma from stent placement^15^^,^^16^.

Although more recent studies do not report any increase in recurrence rate^17–19^, the literature on the impact of stents on cancer recurrence is partly contradictory^12^^,^^20^^,^^21^. Accumulating data indicate that SEMS are associated with a decreased incidence of postoperative complications and lower stoma rate^17^^,^^18^^,^^21–26^, whereas data on long-term outcomes are scarce but point to similar long-term survival^19^^,^^26^^,^^27^. Neither NICE (2014) nor the Cochrane Collaboration provide any firm conclusion on the use of SEMS as a bridge to surgery but state that it is an alternative that could be considered^28^^,^^29^. However, in the NICE guidelines updated in 2020, SEMS as a bridge to surgery is considered a good alternative to direct surgery in patients presenting with acute left-sided large bowel obstruction^30^. Also, the European Society of Gastrointestinal Endoscopy guidelines have shifted from advocating not to use SEMS as a bridge to surgery in fit patients (ASA II or less) aged <75 years^31^^,^^32^ to consider it as a treatment option in patients with potentially curable left-sided obstructing colon cancer as an alternative to emergency resection^33^. The evidence base is, however, still not solid, and there is a need for more knowledge, especially on long-term oncological results of SEMS as bridge to surgery^34^. Further, studies comparing SEMS with stomas as means of bridge to surgery are scarce.

This study aimed to compare decompression with stoma or SEMS as a bridge to surgery in malignant large bowel obstruction with focus on long-term oncological outcomes and perioperative morbidity and mortality. Primary endpoints were 5-year overall survival and 3-year disease-free survival. Secondary endpoints were 30-day morbidity and mortality rates, recurrence rate, duration of stay in hospital and stoma rate at 3 years.

## Methods

### Study population and eligibility criteria

In this retrospective nationwide population-based study, the patients were identified via the Swedish Colorectal Cancer Register (SCRCR); the registry had a coverage of 94–99 per cent^2^ during the study period. All patients with acute large bowel obstruction from 1 January 2007 to 31 December 2009, registered as undergoing preoperative (preresection surgery) decompression with a temporary intention (SEMS or stoma) were identified and included. Medical files including physician’s notes and endoscopy examinations were collected from the hospitals from the date of diagnosis until 31 December 2012, including the mandatory 3-year follow-up after resection surgery.

The inclusion criterion was decompression with bridge to surgery intention due to malignant large bowel obstruction. Patients with prophylactic, palliative or other decompression intentions were excluded. Patients with bowel perforation at diagnosis were also excluded.

The following data were retrieved: age, sex, ASA score, preoperative staging, tumour localization, pathology reports including TNM stage, types of complications, readmission, stoma at 3 years, recurrence and death of any cause. The date of death was obtained from the Swedish Population Register and the follow-up duration was 5 years for each patient. The patients were divided into two groups (SEMS and stoma), and analyses were performed per protocol.

Ethical approval for this study was obtained from the Regional Ethics Committee in Lund, dnr 2010/260.

### Endpoints

The primary endpoints were 5-year overall survival (OS) and 3-year disease-free survival. OS and disease-free survival were calculated from the date of resection surgery until recurrent disease, death or end of follow-up in all cases that underwent subsequent resection. In cases that did not undergo subsequent resection OS and disease-free survival were calculated from the date of diagnosis. In the analysis of disease-free survival, all patients not radically operated (macroscopically and microscopically) were excluded. Disease-free survival was compared between groups by calculating the risk of recurrent disease.

Secondary endpoints were 30-day morbidity and mortality rates, recurrence rate (locoregional and distant), duration of stay in hospital and stoma rate at 3 years.

The Clavien–Dindo classification (CD)^35^, was used to compare morbidity. Briefly, the classification was used to differentiate complications as follows: CD 2 (minor complication only requiring medical attention); CD 3a (complication requiring surgical intervention but not general anaesthesia); CD 3b (complication requiring surgical intervention under general anaesthesia); and CD 4 (complication leading to organ failure). CD scores were not in the registry and therefore calculated based on the information in the medical files and registry data.

Recurrence rate analyses were, as in disease-free survival, based on radical operations only.

Finally, a subgroup analysis was conducted focused on left-sided tumours only.

### Statistical analysis

The Pearson χ^2^ test was used to compare categorical variables, and the Mann–Whitney U test was used to compare continuous variables. In the 30-day mortality rate, Fisher’s exact test was used. Survival analysis data were measured from the day of resection surgery to death of any cause. In patients with a bridge-to-surgery intention in whom resection surgery was never performed, the day of decompression was used. The Kaplan–Meier method was used in the survival analyses. As the Kaplan–Meier curves showed that the assumption of proportional hazards was not fulfilled, survival was first analysed using logistic regression analyses. The follow-up duration was thus divided into two periods, enabling performance of Cox regression analyses. Multivariable analyses adjusting for potential confounders were performed in the two groups. Missing values were coded as a separate category.

Analyses were performed using IBM SPSS^®^ version 23, Armonk, NY, USA. Differences were considered statistically significant at *P* < 0.050.

## Results

**Fig. 1:**
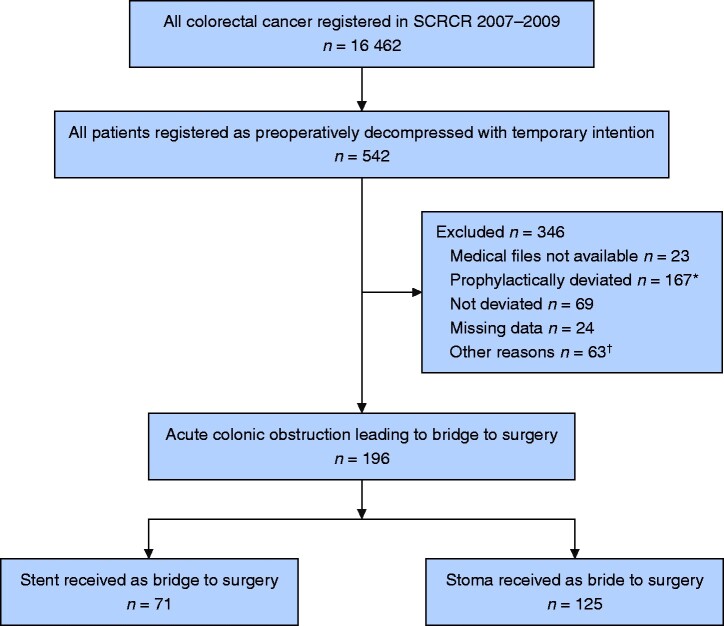
Flow chart diagram *Refers to planned decompression before start of neoadjuvant chemotherapy and/or radiotherapy. ^†^Non-colonic obstruction, palliative decompression, other. SCRCR, Swedish Colorectal Cancer Register

A total of 542 patients in 52 different hospitals were registered as undergoing preoperative decompression with a temporary intention, of which it was possible to retrieve medical files for 519 cases. A total of 196 patients met the inclusion criteria (SEMS, 71 patients, stoma, 125 patients). All patients who lacked verification of acute malignant obstruction were excluded (*Fig. 1*).

Of the 52 hospitals, 15 did not use bridge to surgery at all, 18 centres used only stoma for decompression and 19 used both SEMS and stoma. Of these 19 centres only three were able to use SEMS off-office time. In the centres using SEMS, a total of 136 cases were treated, of which 71 were treated with SEMS and 65 with stoma, whereas the remaining 60 cases with stoma were treated in centres only using stoma. Statistically the SEMS and stoma groups did not differ significantly in sex, age, ASA score and TNM stage (*Table 1*), although a borderline significant difference towards higher age was noted in the SEMS group (median 72 (range 41–91) years *versus* 66 (range 36–91) years, *P* = 0.060). Significantly more rectal tumours had a stoma (35 per cent) than SEMS (13 per cent). No difference was found in the proportion of patients referred to adjuvant treatment (34 per cent in the SEMS group *versus* 40 per cent in the stoma group) (*Table 1*). For adjuvant treatment, 5-fluorouracil intravenously or orally with or without concomitant oxaliplatin were used. No patient was treated with biological agents including Bevacizumab.

**Table 1:** Baseline characteristics

	**SEMS** **(*n* = 71)**	**Stoma** **(*n* = 125)**	*P*
**Sex**			
Female	31 (44)	53 (42.4)	
Male	40 (56)	72 (57.6)	0.860^§^
Age (years)*	72 (41–91)	66 (36–91)	0.080^¶^
**ASA classification**			
ASA I	12 (17)	18 (14.4)	
ASA II	37 (52)	67 (53.6)	
ASA III	17 (24)	33 (26.4)	
ASA IV	4 (6)	5 (4.0)	
Missing	1 (1)	2 (1.6)	0.900^§^
**TNM stage**			
T2	3 (4)	6 (4.8)	
T3	49 (69)	68 (54.4)	
T4	17 (24)	40 (32.0)	
Tx	1 (1)	2 (1.6)	
N/A^†^	1 (1)	9 (7.2)	0.480^§^
N0	30 (42)	60 (48.0)	
N1	25 (35)	29 (23.2)	
N2	15 (21)	26 (20.8)	
Nx	1 (1)	1 (0.8)	
N/A^†^	0 (0)	9 (7.2)	0.460^§^
M0	54 (76)	96 (76.8)	
M1	15 (21)	25 (20.0)	
Mx	1 (1)	2 (1.6)	
Missing	1 (1)	2 (1.6)	0.980^§^
**Complete preoperative staging** ^‡^			
Yes	55 (77)	105 (84.0)	
No	16 (23)	20 (16.0)	0.260^§^
**Tumour localization**			
Colon	62 (87)	81 (64.8)	
Rectum	9 (13)	44 (35.2)	<0.010^§^
**Adjuvant chemotherapy**			
Yes	24 (34)	46 (40.4)	
No	45 (64)	68 (59.6)	
Missing	1 (1)	0 (0.0)	0.450^§^

Values in parentheses are percentages unless indicated otherwise;

* values are median (range).

† Patients not operated.

‡ Preoperative screening for metastases by CT scan of the abdomen and thorax with intravenous contrast.

§ Pearson χ^2^ test;

¶ Mann–Whitney U test. N/A, not available.

One patient (1 per cent) in the SEMS group did not undergo resection surgery compared to 11 (8.8 per cent) patients in the stoma group (*P* = 0.040). In the SEMS group, a patient who received neoadjuvant treatment developed bowel ischaemia after 3 months. In the stoma group, three patients did not undergo resection surgery due to complications after the initial stoma procedure (one patient had recurrent postoperative pneumonia and died after 2 months, two patients had stoma failure with subsequent infections and died after 1.5 months and 2 months), and eight patients were converted to palliative treatment due to tumour progression (five of which were during neoadjuvant treatment).

### Primary endpoints

**Fig. 2:**
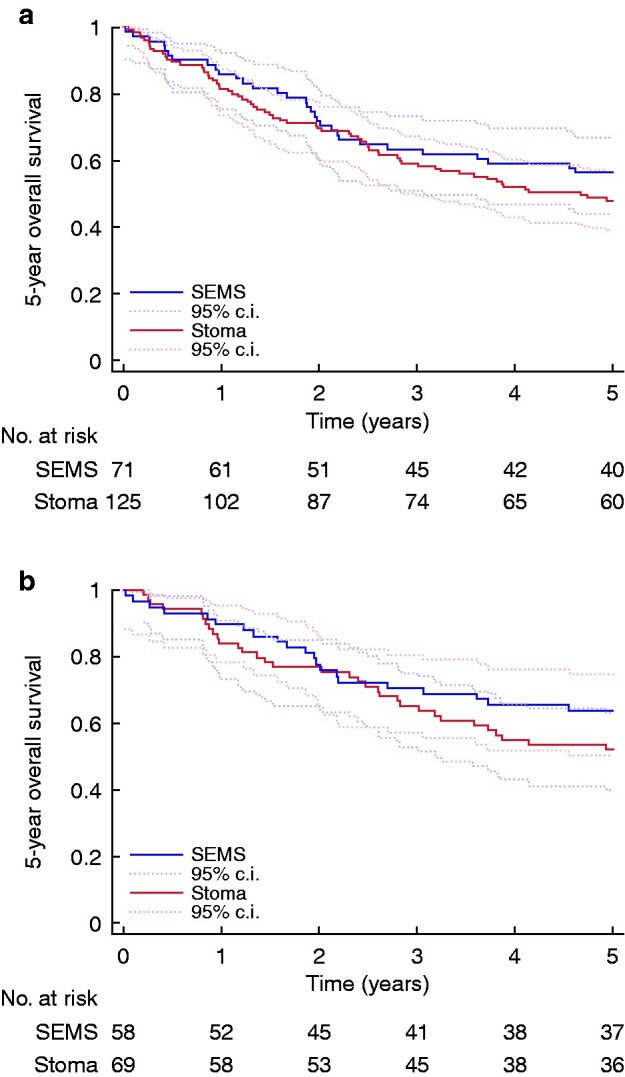
Kaplan–Meier plots of 5-year overall survival **a** All tumours. **b** Left-sided tumours. SEMS, self-expanding metallic stent

The 5-year OS did not differ significantly between groups: 56 per cent (40 of 71 patients) in the SEMS group compared with 48 per cent (60 of 125) in the stoma group (*P* = 0.260). Moreover, no statistical difference in the 3-year disease-free survival was noted (SEMS, 73 per cent (43 of 59 patients); stoma, 65 per cent (62 of 95), *P* = 0.320). Kaplan–Meier curves are shown in *Fig. 2a*. After adjusting for age, stage, sex and ASA score in the multivariable analyses, no significant difference was noted in the 5-year survival by logistic regression analyses (*Table 2*). In the Cox regression analyses however, a significantly better survival rate was noted in the SEMS group in the second half of the study period (2.5–5 years).

**Table 2:** Survival analyses

			Univariable		Multivariable	
Logistic regression	Patients	Dead*	OR (95% c.i.)	OR (95% c.i.)†	OR (95% c.i.)‡	OR (95% c.i.)§
**A. Overall survival – risk of death**						
**OS at 5 years**						
SEMS	71	31 (44)	1.00	1.00	1.00	1.00
Stoma	125	65 (52.0)	1.40 (0.78–2.51)	1.61 (0.86–3.03)	1.59 (0.77–3.32)	1.42 (0.67–3.02)
**OS 0–2.5 years interval**						
SEMS	71	25 (35)	1.00	1.00	1.00	1.00
Stoma	125	44 (35.2)	1.04 (0.63–1.69)	1.06 (0.64–1.76)	0.97 (0.56–1.66)	0.85 (0.48–1.48)
**OS 2.5–5 years interval**						
SEMS	46	6 (13)	1.00	1.00	1.00	1.00
Stoma	81	21 (26)	2.16 (0.87–5.36)	2.21 (0.86–5.67)	3.51 (1.25–9.87)	3.46 (1.18–10.1)
**B. Disease-free survival – risk of recurrence in radically resected patients**						
**COX regression**	**Patients**	Recurrent disease*	**HR (95% c.i.)**	**HR (95% c.i.)** ^†^	**HR (95% c.i.)** ^‡^	**HR (95% c.i.)** ^§^
**DFS at 3 years**						
SEMS	59	16 (27)	1.00	1.00	1.00	1.00
Stoma	95	33 (35)	1.43 (0.70–2.92)	1.34 (0.65–2.79)	1.77 (0.77–4.10)	1.88 (0.80–4.45)
**COX regression**	**Patients**	Recurrent disease*	**HR (95% c.i.)**	**HR (95% c.i.)** ^†^	**HR (95% c.i.)** ^‡^	**HR (95% c.i.)** ^§^
**DFS 0–3 years**						
SEMS	59	16 (27)	1.00	1.00	1.00	1.00
Stoma	95	33 (35)	1.36 (0.75–2.47)	1.31 (0.71–2.41)	1.46 (0.77–2.78)	1.56 (0.80–3.04)

*Values in parentheses are percentages.

† Adjusted for age at diagnosis, sex and ASA class.

‡ Adjusted for age at diagnosis, sex, ASA class and TNM stage.

§ Adjusted for age at diagnosis, sex, ASA class, TNM stage and tumour site (colon/rectum). OR, odds ratio; HR hazard ratio; OS, overall survival; SEMS, self-expanding metallic stent; DFS, disease-free survival.

### Secondary endpoints

As shown in *Table 3*, 184 patients (94 per cent) underwent resection surgery, of which 83 per cent (59 of 71 patients) in the SEMS group and 76 per cent (95 of 125) in the stoma group were radically resected (macro- and microscopically).

**Table 3:** Secondary endpoints

Oncological endpoints			
	**SEMS**	**Stoma**	***P*** ^¶^
**Radically resected** ^†^	59 of 71 (83)	95 of 125 (76.0)	0.870
**Not radically resected**	11 of 71 (15)	19 of 125 (15.2)	0.870
**Not resected**	1 of 71 (1)	11 of 125 (8.8)	0.040
**Total recurrence**	16 of 59 (27)	33 of 95 (35)	0.320
**Locoregional recurrence**	7 of 59 (12)	12 of 95 (13)	0.890
**Distant recurrence**	15 of 59 (25)	30 of 95 (32)	0.410
**Morbidity endpoints**			
**30-day mortality**	1 of 71 (1)	1 of 125 (0.8)	1.000^#^
**Complications**			
**Decompression surgery**			
Total^‡^	17 of 71 (24)	45 of 125 (36.0)	0.080
Severe^§^	10 of 71 (14)	21 of 125 (16.8)	0.620
**Resection surgery**			
Total^‡^	24 of 70 (34)	33 of 114 (28.9)	0.450
Severe^§^	15 of 70 (21)	13 of 114 (11.4)	0.070
**Decompression and resection surgery combined**			
Total^‡^	36 of 71 (25)	65 of 125 (36.8)	0.860
Severe^§^	23 of 71 (21)	32 of 125 (19.2)	0.310
**Length of hospital stay (days)**			
Decompression surgery^*^	4 (1–6)	9 (7–13)	<0.010^**^
Resection surgery^*^	8 (6–11)	8 (7–13)	0.160**
Combined^*^	12 (9–18)	17.5 (14–23)	<0.010^**^
**Readmissions**			
30 days	3 of 70 (4)	16 of 123 (13.0)	0.050^¶^
365 days	21 of 68 (31)	49 of 110 (44.5)	0.07
**Remaining stoma at 3 years**	5 of 45 (11)	20 of 71 (28)	0.030

Values in parentheses are percentages unless indicated otherwise;

* values are median (i.q.r.).

† Macroscopically and microscopically.

‡ Clavien–Dindo classification 2–5.

§ Clavien–Dindo 3b–5.

¶ Pearson χ^2^ test, except

# Fisher’s exact test,

** Mann–Whitney U test.

The total recurrence rate did not differ between groups: 27 per cent (16 of 59 patients) in the SEMS group and 35 per cent (33 of 95) in the stoma group. Moreover, no difference in locoregional and distant recurrences was found between groups (*Table 3*).

No difference in 30-day mortality was noted between the groups: SEMS, 1 per cent (1 of 71 patients), and stoma, 0.8 per cent (1 of 125).

There was no significant difference in complication rate during decompression between the SEMS and stoma groups. The total complication rate during decompression (CD 2–5) and severe complication rate (CD 3b–5) were 24 and 14 per cent in the SEMS group and 36 and 17 per cent in the stoma group, respectively (*Table 3*). The total complication rates during resection surgery were 34 and 29 per cent in the SEMS and stoma groups, respectively. A borderline difference towards more severe complications during resection surgery was noted in the SEMS group (21 per cent) compared to the stoma group (11 per cent) (*P* = 0.070). The combined complication rates (decompression and resection) did not differ between the groups.

Stent perforations occurred in six of 71 patients (8 per cent), of which four were in-hospital and two were late perforations. None of the patients were treated with chemotherapy or biological agents before or at the time of perforation. No mortality directly related to perforation was noted, and there was no difference in 5-year OS (50 per cent (3 of 6 patients) in SEMS with perforation and 57 per cent (37 of 65 patients) in SEMS with no perforation; *P* = 0.740).

The duration of hospital stay for decompression procedure was significantly lower in the SEMS group with a median of 4 days compared with 9 days in the stoma group. No difference was found for resection surgery with a median of 8 days in each group. The aggregated median duration of stay for both procedures was 12 (i.q.r. 9–18) days in the SEMS group and 17.5 (i.q.r. 14–23) days in the stoma group (*P* < 0.010).

The 30-day readmission rate was lower in the SEMS group (4 per cent, 3 of 70 patients) compared to that in the stoma group (13 per cent, 16 of 123) (*P* = 0.050). A borderline difference towards a lower readmission rate at 365 days was noted in the SEMS group (31 per cent, 21 of 68 patients) compared to that in the stoma group (45 per cent, 49 of 110) (*P* = 0.070).

The permanent stoma rate was lower in the SEMS group (11 per cent, 5 of 45 patients) than in the stoma group (28 per cent, 20 of 71) (*P* = 0.030).

### Subgroup analyses of left-sided tumours

Of the total 196 patients treated as bridge to surgery, 127 cases were due to left-sided colon tumours. Of these, 46 per cent (58 patients) were treated by SEMS and 54 per cent (69 patients) were operated with a stoma. Tumour and patient characteristics did not differ between groups and were similar to the main study cohort.

**Fig. 3:**
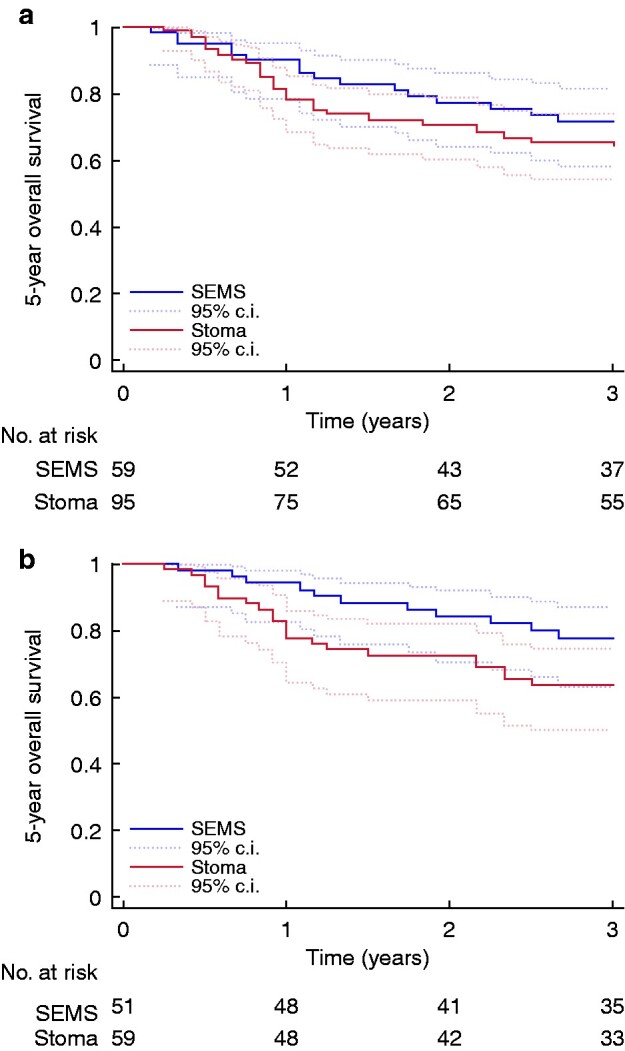
Kaplan–Meier plots of 3-year disease-free survival **a** All tumours. **b** Left-sided tumours. SEMS, self-expanding metallic stent

Five-year OS did not differ statistically significantly between the groups: 64 per cent (37 of 58 patients) in the SEMS group compared to 52 per cent (36 of 69) in the stoma group (*P* = 0.230), nor did 3-year disease-free survival: SEMS 78 per cent (40 of 51 patients); stoma 64 per cent (38 of 59), *P* = 0.090. Kaplan–Meier curves are shown in *Figs 2b* and *3b*. Logistic regression showed an odds ratio (OR) of 2.28 (95 per cent c.i. 0.88 to 5.90) in 5-year overall survival (*P* = 0.090) and 2.62 (95 per cent c.i. 0.94 to 7.32) in 3-year disease-free survival (*P* = 0.070). As in the total population, the multivariable Cox regression analyses (adjusted for age, stage, sex and ASA score) showed no statistically significant difference in OS the first 2.5 years, hazard ratio (HR) 1.18 (95 per cent c.i. 0.56 to 2.48) but did in the 2.5–5 year period, HR 5.74 (95 per cent c.i. 1.41 to 23.38). A hazard ratio of 2.19 (95 per cent c.i. 0.98 to 4.93) in 3-year disease-free survival was noted (*P* = 0.060) (*Appendix S1*).

## Discussion

This large registry-based study showed no statistically significant difference in 5-year OS or 3-year disease free survival by SEMS treatment compared to decompression with stoma as bridge to surgery in malignant large bowel obstruction. No differences in the rate of locoregional recurrences or distant metachronous metastases were noted; moreover, SEMS treatment was associated with a significantly shorter duration of hospital stay and a lower rate of both temporary and permanent stomas. Also, the subgroup analyses of left-sided tumours provided similar results with regard to OS, disease-free survival and morbidity. The results indicate that the use of SEMS as a bridge to surgery is a safe procedure in the management of malignant large bowel obstruction with some advantages compared with decompression by stoma.

Many early studies on the role of SEMS in acute malignant obstruction were conducted on small or heterogeneous populations including palliative and curative cases. The Dutch RCT^11^ indicated a high risk of complications, including perforations, while another study reported an increased risk of local recurrences following the use of SEMS^14^; on this basis, several guidelines did not recommend the use of SEMS in potentially curative cases. In Sweden, the use of SEMS in patients without metastasis has recently declined to only two to five patients per year^2^ compared to 18–29 per year during the study period. Risk factors for SEMS failure have later been identified, such as balloon dilatation and covered stents^36^^,^^37^, leading to improved results with early perforation rates of less than 5 per cent in specialized centres^38^^,^^39^. Even though perforation might not be a frequent problem, it has been reported to carry a high risk of mortality^38^ and has to be considered. To be noted, none of the patients were treated with chemotherapy or biological agents before or at the time of perforation, in line with the Swedish guidelines at the time of the study period, in which biological agents were not recommended for either neoadjuvant or adjuvant treatment.

Knowledge on the long-term consequences of perforation is extremely limited and contradictory results have reported both limited effect^40^ as well as high risk of recurrences^41^. Although the present study only included six cases with stent perforation, no adverse effect on long-term survival was noted.

Recently, several studies have shown that, in the bridge-to-surgery setting, SEMS is associated with lower rates of perioperative complications and less need for permanent stomas^18^^,^^21–24^^,^^26^^,^^34^^,^^42^. Accumulating data also indicate that, in contrast to early concerns, SEMS seems to be safe, with similar long-term survival to decompression with stoma or emergency resection^27^^,^^43–46^, and there have been encouraging early results provided by two randomized trials^19^^,^^26^. Similar results were also presented in a recent Dutch registry-based study, although with a shorter follow-up duration^47^. Furthermore, there are reports indicating that a bridge-to-surgery strategy is superior to emergency resection^48^^,^^49^. The results of the present study are in line with these recent studies and meta-analyses, although straight comparisons between SEMS and stoma are scarce.

This study has some limitations. It is in essence a retrospective study, albeit partially based on prospectively collected registry data. The design entails a risk of selection bias when performing group comparisons. However, no significant differences in demography between the groups were noted, with the exception of tumour location and a tendency towards older age in the SEMS group, and the results were stable after adjusting for age, tumour location, sex, ASA score and TNM stage in the multivariable analyses. Although all medical files were thoroughly scrutinized, it was not possible to track the reasons behind the selected decompression method in the centres using both methods. SEMS was used in only half of the centres utilizing the bridge-to-surgery concept and was not available off-office time in the vast majority of these institutions (16 centres, 84 per cent). Thus, the allocation of patients could depend predominately on availability for SEMS. Besides availability, technical aspects are most likely to be the key factor in the choice of method but are less likely to influence mortality. One problem is that the number of cases where SEMS were intended but failed to be placed was not available. Thus, it was not possible to draw any firm conclusions on success rate or complete procedure-related complications for SEMS deployment, and as such, the study is to be regarded as a per-protocol analysis.

On the other hand, the main strength is the long-term follow-up of the population-based, nationwide, well defined material solely constituting cases of acute malignant large bowel obstruction managed with a bridge-to-surgery approach.

This study documented at least equivalent long-term survival and similar local and distant recurrence rates with the use of SEMS compared with stoma as a bridge to surgery in acute malignant large bowel obstruction. Moreover, SEMS was associated with shorter hospital stay, fewer permanent stomas and a higher rate of fulfilled resection surgery. Taken together, these data should encourage a change in the predominant approach and increase the use of SEMS as a bridge to surgery in malignant large bowel obstruction.

## Funding

This study was supported by Skåne County Counciĺs Research and Development Foundation grants, ALF-agreement (2018-Project 0264) between the Swedish government and the county councils, and local grant (Allmänna Sjukhusets i Malmö Stiftelse för bekämpande av cancer).

*Disclosure.* The authors declare no conflict of interest.

## Supplementary material

Supplementary material is available at *BJS Open* online.

## Supplementary Material

zrab018_Supplementary_Data

## Notes

Preliminary results were, in part, presented as a poster at the 12^th^ Scientific and Annual Meeting of the European Society of Coloproctology, 20–22 September 2017, CityCube, Berlin, Germany
