# Corrigendum to: Long-term survival after self-expanding metallic stent or stoma decompression as bridge to surgery in acute malignant large bowel obstruction

**DOI:** 10.1093/bjsopen/zrab066

**Published:** 2021-08-19

**Authors:** T Axmarker, M Leffler, M Lepsenyi, H Thorlacius, I Syk

*BJS Open*, https://doi.org/10.1093/bjsopen/zrab018

When this paper first published, in fig. 3, both graphs erroneously used the text “5-year overall survival” in their y axes. This should have read “3-year disease-free survival”. This has now been corrected online.

