# Supplementary material for: Long-term survival after self-expanding metallic stent or stoma decompression as bridge to surgery in acute malignant large bowel obstruction
Source: BJS Open. 2021 Apr 21;5(2):zrab018. doi: 10.1093/bjsopen/zrab018 (PMC8058149; doi:10.1093/bjsopen/zrab018)
Supplement: zrab018_Supplementary_Data [file zrab018_supplementary_data.docx]

**Appendix 1 - Survival analyses left sided tumors**

|  |  |  |  |  |  |
| --- | --- | --- | --- | --- | --- |
|  |  |  | **Univariate** | **Multivariate** | |
|  |  |  |  |  |  |
| **Logistic regression** | **Patients** | **Dead** | **OR** (CI 95%) | **OR** (CI 95%)^a^ | **OR** (CI 95%)^b^ |
|  | n | n (%) |  |  |  |
| **OS at 5 years** |  |  |  |  |  |
| Stent | 58 | 21 (36.2%) | 1.00 | 1.00 | 1.00 |
| Stoma | 69 | 33 (47.8%) | 1.62 (0.79-3.30) | 2.07 (0.90-4.72) | 2.28 (0.88-5.90) |
|  |  |  |  |  |  |
| **COX regression** | **Patients** | **Dead** | **HR** (CI 95%) | **HR** (CI 95%)^a^ | **HR** (CI 95%)^b^ |
|  | n | n (%) |  |  |  |
| **OS < 2,5 years interval** |  |  |  |  |  |
| Stent | 58 | 16 (27.6%) | 1.00 | 1.00 | 1.00 |
| Stoma | 69 | 20 (29.0%) | 1.08 (0.56-2.08) | 1.27 (0.64-2.52) | 1.18 (0.56-2.48) |
|  |  |  |  |  |  |
| **OS 2,5 - 5 years interval** |  |  |  |  |  |
| Stent | 42 | 5 (11.9%) | 1.00 | 1.00 | 1.00 |
| Stoma | 49 | 13 (26.5%) | 2.43 (0.87-6.83) | 2.82 (0.92-8.65) | 5.74 (1.41-23.38) |
|  |  |  |  |  |  |
|  |  |  |  |  |  |
|  |  |  |  |  |  |
|  |  |  | **Univariate** | **Multivariate** | |
|  |  |  |  |  |  |
| **Logistic regression** | **Patients** | **Recurrent disease** | **OR** (CI 95%) | **OR** (CI 95%)^a^ | **OR** (CI 95%)^b^ |
|  | n | n (%) |  |  |  |
| **DFS at 3 years** |  |  |  |  |  |
| Stent | 51 | 11 (21.6%) | 1.00 | 1.00 | 1.00 |
| Stoma | 59 | 21 (35.6%) | 2.01 (0.86-4.72) | 1.95 (0.81-4.71) | 2.62 (0.94-7.32) |
|  |  |  |  |  |  |
| **COX regression** | **Patients** | **Recurrent disease** | **HR** (CI 95%) | **HR** (CI 95%)^a^ | **HR** (CI 95%)^b^ |
|  | n | n (%) |  |  |  |
| **DFS 0 - 3 years** |  |  |  |  |  |
| Stent | 51 | 11 (21.6%) | 1.00 | 1.00 | 1.00 |
| Stoma | 59 | 21 (35.6%) | 1.87 (0.90-3.87) | 1.84 (0.87-3.88) | 2.19 (0.98-4.93) |
|  |  |  |  |  |  |

a Adjusted for age at diagnosis, sex and ASA-class

b Adjusted for age at diagnosis, sex, ASA-class and TNM-classification
